# Eicosapentaenoic and docosahexaenoic acids-rich fish oil supplementation attenuates strength loss and limited joint range of motion after eccentric contractions: a randomized, double-blind, placebo-controlled, parallel-group trial

**DOI:** 10.1007/s00421-016-3373-3

**Published:** 2016-04-16

**Authors:** Yosuke Tsuchiya, Kenichi Yanagimoto, Koichi Nakazato, Kohsuke Hayamizu, Eisuke Ochi

**Affiliations:** Laboratory of Health and Sports Sciences, Meiji Gakuin University, Kanagawa, Japan; Human Life Science R&D Center, Nippon Suisan Kaisha, Ltd., Tokyo, Japan; Graduate School of Health and Sport Science, Nippon Sport Science University, Tokyo, Japan; General Health Medical Center, Yokohama University of Pharmacy, Kanagawa, Japan; Faculty of Bioscience and Applied Chemistry, Hosei University, Tokyo, Japan

**Keywords:** Eicosapentaenoic acid, Docosahexaenoic acids, Lengthening contractions, Supplement, Muscle dysfunction, Muscle strength

## Abstract

**Purpose:**

This study investigated the effect of eicosapentaenoic and docosahexaenoic acids-rich fish oil (EPA + DHA) supplementation on eccentric contraction-induced muscle damage.

**Methods:**

Twenty-four healthy men were randomly assigned to consume the EPA + DHA supplement (EPA, *n* = 12) or placebo (PL, *n* = 12) by the double-blind method. Participants consumed EPA + DHA or placebo supplement for 8 weeks prior to exercise and continued it until 5 days after exercise. The EPA group consumed EPA + DHA-rich fish oil containing 600 mg EPA and 260 mg DHA per day. Subjects performed five sets of six maximal eccentric elbow flexion exercises. Changes in the maximal voluntary contraction (MVC) torque, range of motion (ROM), upper arm circumference, muscle soreness as well as serum creatine kinase, myoglobin, IL-6, and TNF-α levels in blood were assessed before, immediately after, and 1, 2, 3, and 5 days after exercise.

**Results:**

MVC was significantly higher in the EPA group than in the PL group at 2–5 days after exercise (*p* < 0.05). ROM was also significantly greater in the EPA group than in the PL group at 1–5 days after exercise (*p* < 0.05). At only 3 days after exercise, muscle soreness of the brachialis was significantly greater in the PL group than in the EPA group (*p* < 0.05), with a concomitant increase in serum IL-6 levels in the PL group.

**Conclusion:**

Eight-week EPA + DHA supplementation attenuates strength loss and limited ROM after exercise. The supplementation also attenuates muscle soreness and elevates cytokine level, but the effect is limited.

## Introduction

Resistance exercise performed two to three times per week to increase muscle strength and mass is recommended by the American College of Sports Medicine (Garber et al. 2011). However, excessive eccentric contractions (ECCs) cause the development of delayed-onset muscle soreness (DOMS), the reduction of maximal strength, limitation of range of motion (ROM), and muscle swelling (Clarkson et al. 1992). DOMS has been shown to peak 1–3 days after ECC before resolving after approximately 1 week (Chen et al. 2011). DOMS is one of the symptoms of muscle damage and demonstrates no relationship between DOMS and other indicators of muscle damage such as MVC, ROM, circumference, and serum CK) (Nosaka et al. 2002).

ECC-induced muscle damage is defined as morphological changes in the sarcomeres and endomysium and inflammatory responses in muscle fibers and connective tissues (Peake et al. 2005). Increase in serum myoglobin (Mb) and creatine kinase (CK) levels have been shown to be associated with damaged muscle tissue (Clarkson et al. 1992). In addition, a number of inflammatory cytokines, including tumor necrosis factor-α (TNF-α) and interleukin-6 (IL-6), are produced in response to ECCs (Clarkson et al. 1992; Ostrowski et al. 1999; Phillips et al. 2003). TNF-α is released from damaged tissue, which appears to relate to inflammation (Dennis et al. 2004). In addition, IL-6 is a known trigger of anti-inflammatory cytokines, and exercise-induced IL-6 is also defined as anti-inflammatory cytokine and shows improved insulin resistance after exercise (Pedersen and Fischer 2007; Panza et al. 2015).

As fish oils containing rich omega-3 polyunsaturated fatty acids are known to have anti-inflammatory effects, they were expected to inhibit muscle damage following exercise (Gray et al. 2014; Jouris et al. 2011; Tartibian et al. 2011). Eicosapentaenoic acid (EPA) and docosahexaenoic acid (DHA) are major fatty acids classified as omega-3 polyunsaturated fatty acids. Numerous scientists have reported the various physiological functions of EPA and DHA including lipid metabolism, anti-inflammation, and cognitive function (Calder 2015; Eslick et al. 2009; Jiao et al. 2014).

Previous studies have reported the efficacy of EPA and DHA supplementation in reducing exercise-induced limited ROM, development of DOMS, muscle swelling, increase in serum CK, and IL-6 (DiLorenzo et al. 2014; Houghton and Onambele 2012; Jouris et al. 2011; Tartibian et al. 2009, 2011). In a review article, it has been suggested that an ingestion ratio of EPA to DHA of approximately 2:1 may be beneficial in counteracting exercise-induced inflammation (Mickleborough 2013). Taribian et al. (2009, 2011) examined the effects of 324 mg/day EPA and 216 mg/day DHA ingestion for 30 days on muscle damage after 40 min of bench stepping. Serum levels of TNF-α after 324 mg/day EPA and 216 mg/day DHA induced (Tartibian et al. 2011) development of DOMS, limited ROM, and significantly inhibited muscle swelling (Tartibian et al. 2009) in response to this regimen. From these studies, it appears that EPA and DHA attenuate inflammatory cytokines, limited ROM, and increased DOMS, CK, and Mb. However, there is no study to show the positive effects of EPA and DHA on decrease in muscle strength after strenuous exercise. Lenn et al. (2002) reported that ingestion of 400 mg/day EPA and 270 mg/day DHA for 30 days did not reduce the decrease in muscle strength. Taken together, more than 400 mg/day EPA and 200 mg/day DHA may be needed to show the beneficial effect on exercise-induced muscle damage. On the other hand, the amount of EPA and DHA is limited to a total of 3000 mg per day for safety in humans by the natural medicines comprehensive database (US Food and Drug Administration 2000). From the previous studies and considering the safety factor, we thought that ingestion of 400–2000 mg/day EPA and 200–1000 mg/day DHA (ratio of EPA to EHA is approximately 2:1) is needed for the attenuation in muscle strength using ECC.

In particular, for the supplementation period, it has been reported that the necessary amounts of EPA and DHA into the myocardium is satisfied by the ingestion of fish oil for 30–60 days (Metcalf et al. 2007). However, previous studies focused on muscle strength for the period of 21–30 days (DiLorenzo et al. 2014; Houghton and Onambele 2012; Lenn et al. 2002). In addition, in the majority of the previous studies, the effects of omega-3 consumption on exerted torque and work output during exercise are uncertain. Therefore, there is a need for studies that evaluate longer periods and larger doses of omega-3 polyunsaturated fatty acid supplementation for muscular strength, including other muscle damage markers after simple joint movement by quantifying the exerted torque and work output.

We hypothesized that 600 mg/day EPA and 260 mg/day DHA for 8 weeks would attenuate the maximal strength in response to ECC. The aim of the present study was to investigate the efficacy of EPA and DHA-rich fish oil supplementation for 8 weeks in reducing ECC-induced muscle damage, including development of DOMS, limit of ROM, muscle swelling, and increased serum levels of inflammatory cytokines and muscle damage-related proteins.

## Methods

### Subjects

A total of 24 Japanese healthy men (age, 19.5 ± 0.8 years; height, 174.4 ± 6.0 cm; weight, 65.3 ± 7.7 kg; body fat, 13.3 ± 3.1 %) were recruited for this study. They were allergic to fish and resistance training and requested to avoid participation in other clinical trials and interventions such as massage, stretching, strenuous exercise, excessive consumption of food or alcohol, and any supplement or medication during the experimental period. All participants were provided with detailed explanations of the study protocol prior to participation and signed an informed consent form in accordance with the Declaration of Helsinki before being enrolled in this study. This study was approved by the Ethics Committee for Human Experiments at Juntendo University (ID: 26–113) and has been registered at the University Hospital Medical Information Network Clinical Trials Registry (UMIN-CTR, identifier: UMIN000016149). The estimation was based on the effect size of 1, alpha level of 0.05, and a power (1−b) of 0.80 for the comparison EPA group and PL group following ECC, which showed that at least ten participants were necessary (G*power, version 3.0.10).

### Study design

The study was performed by a double-blind, placebo-controlled, parallel-group trial design. All subjects were randomly assigned to two groups in such a manner as to minimize the inter-group differences in age, body fat, BMI, and maximal voluntary isometric contraction (MVC) torque, and they consumed either EPA and DHA (EPA, *n* = 12; age, 19.4 ± 0.7 years; height, 174.4 ± 5.6 cm; weight, 64.3 ± 7.7 kg; body fat, 13.0 ± 3.5 %; MVC, 58.6 ± 14.9 Nm) or placebo (PL, *n* = 12; age, 19.5 ± 0.8 years; height, 174.3 ± 6.7 cm; weight, 66.2 ± 8.0 kg; body fat, 13.6 ± 2.8 %; MVC, 58.0 ± 11.6 Nm) for 8 weeks prior to the exercise experiment and continued it until 5 days after the exercise. Sequence allocation concealment and blinding to subjects and researchers were maintained during treatment. Since it has been reported that the compensation of EPA and DHA concentration into the myocardium is needed for the ingestion of fish oil for 30–60 days (Metcalf et al. 2007), the duration of the total ingestion period was 62 days (including the exercise day). On the day of exercise testing, MVC torque, range of motion of the elbow joint (ROM), upper arm circumference, muscle soreness assessed by a visual analog scale (VAS), and fasting blood samples were measured in the non-dominant arm before exercise. Immediately after baseline measurements, ECC in the non-dominant arm as with the other measurement was performed in each subject. All measurements were performed immediately before, immediately after, and 1, 2, 3, and 5 days after exercise. Measurements were performed in a room maintained at 24−26 °C. In addition, we surveyed the nutrition status of all subjects prior to supplement consumption (8 weeks before the exercise experiment) and after experimental testing of the food frequency questionnaire based on food groups (FFQg, version 3.5, Kenpakusha, Tokyo, Japan). The outcome measures were MVC torque, ROM, upper arm circumference, muscle soreness, and blood serum markers. In addition, we also measured serum fatty acids including EPA, DHA, arachidonic acid (AA), and dihomo-gamma-linolenic acid (DGLA). Based on the two baseline measures of MVC torque, ROM, upper arm circumference, and muscle soreness (before exercise and different day), the test–retest reliability of the measures was examined.

### Supplements

The EPA group consumed eight 300 mg EPA and 130 mg DHA-rich fish oil softgel capsules (Nippon Suisan Kaisha Ltd., Tokyo, Japan) per day (containing 600 mg EPA and 260 mg DHA in a total of 2400 mg). The PL group consumed eight 300 mg corn oil softgel capsules (Nippon Suisan Kaisha Ltd., Tokyo, Japan) per day (not containing EPA and DHA in a total of 2400 mg). Subjects consumed the supplements 30 min after meals with water.

### Eccentric contractions

Each subject was seated in the chair of an isokinetic dynamometer (Biodex Multi-Joint System 3, NY, USA) with one arm set at a shoulder joint angle of 45° flexion, and the elbow joint aligned with the rotation axis of the isokinetic dynamometer. The lever arm of the isokinetic dynamometer was secured to the wrist of subjects in a supinated position. ECC consisted of five sets of six maximal voluntary eccentric contractions of the elbow flexors at a constant velocity of 30°/s for the range of motion from 90° flexion to 0° (full extension). Subjects were verbally encouraged to maximally resist throughout the ROM for 3 s. The isokinetic dynamometer returned the arm to the 90° flexed position at a constant velocity of 30°/s, providing 3 s passive recovery between contractions. The measurements of torque produced during eccentric contractions were stored in a computer connected to the isokinetic dynamometer, and work output and peak torque were calculated later. These measurements were based on a previous study (Chen et al. 2011).

### Maximal voluntary isometric contraction (MVC) torque

MVC torque was measured on the same apparatus and positioning as described for eccentric exercise. Subjects performed two 5-s MVCs at 90° elbow joint angle with a 15-s rest between contractions. The peak torque of the two contractions was used as the MVC torque as described in our previous study (Tsuchiya et al. 2015). The test–retest reliability of the MVC measures based on coefficient of variation (CV) was 3.9 %.

### Range of motion (ROM) of the elbow joint

To examine the elbow joint ROM, two elbow joint angles (extended and flexed joint angles) were measured using a goniometer (Takase Medical, Tokyo, Japan). The extended joint angle was recorded when subjects attempted to fully extend the joint, with the elbow held by their side and the hand in supination. The flexed joint angle was determined when subjects attempted to fully flex the joint from an equally fully extended position with the hand supinated. ROM was calculated by subtracting the flexed joint angle from the extended joint angle (Tsuchiya et al. 2015). The test–retest reliability of the ROM measures based on CV was 4.2 %.

### Upper arm circumference

Upper arm circumference was assessed at 3, 5, 7, 9, and 11 cm above the elbow joint using a tape measure while subjects were standing with the arms relaxed by their side. The mean value of five measurements was used for analysis (Nosaka et al. 2005). Measurement marks were maintained during the experimental period using a semi-permanent ink marker, and a well-trained investigator took the measurements. The average value of three measurements was used for further analysis. The test–retest reliability of the circumference measurements for the five sites based on CV was 2.2–3.4 %.

### Muscle soreness of elbow flexors

Muscle soreness was assessed using a 100-mm VAS in which 0 indicates ‘‘no pain’’ and 100 is the ‘‘worst pain imaginable’’. Each subject was asked to indicate the pain level on the scale when an investigator palpated the biceps brachii, brachialis, or brachioradialis, respectively, using a thumb while subjects relaxed with their arms in the natural position. All tests were conducted by the same investigator who had been trained to apply the same pressure over time and between subjects. The test–retest reliability of the VAS measures for the three muscles based on CV was 0–4.7 %.

### Blood samples

Subjects fasted for 8 h prior to blood samples being taken from the forearm by a trained doctor. Blood samples were allowed to clot at room temperature (25 °C) and then centrifuged at 3000 rpm for 10 min at 4 °C. Serum was extracted and stored at −20 °C until analysis. The serum levels of CK, Mb, IL-6, TNF-α, and four fatty acids (DGLA, AA, EPA, DHA) were measured. Serum CK levels were measured according to the Japan Society of Clinical Chemistry Standardized Method. Serum Mb and IL-6 levels were measured by chemiluminescence enzyme immunoassay (CLEIA), and serum TNF-α levels were measured using an enzyme-linked immunosorbent assay (ELISA). Serum fatty acid levels were measured using gas chromatography spectrometry (GC). The test–retest reliability of the CK, Mb, IL-6, and TNF-α measurements based on CV was 3.8, 2.8, 2.9, and 11.4 %, respectively. Also, that of the DGLA, AA, EPA, and DHA measurements based on CV were 5.6, 4.9, 5.1, and 6.6 %, respectively.

### Statistical analyses

All analyses were performed using SPSS Statistics software version 20.0 (IBM, Armonk, NY). All variables are expressed as means ± standard deviations (SD). All data were confirmed within the normal distribution by Kolmogorov–Smirnov test. MVC, ROM, upper arm circumference, muscle soreness, and blood samples over time were compared between the PL and EPA groups by a two-way repeated measure analysis of variance (ANOVA). When a significant interaction or time effect was found, Bonferroni’s correction was performed as a post hoc test. A *p* value of <0.05 was considered to be statistically significant.

## Results

Nobody dropped out during this study period. As a result of food frequency questionnaire, no difference in nutrition status was observed between the EPA group (energy, 2578.2 ± 610.7 kcal; protein, 82.0 ± 27.8 g; fat, 88.7 ± 22.2 g; carbohydrate, 348.5 ± 94.3 g; omega3 fatty acid, 2.6 ± 1.4 g) and PL group (energy, 2413.3 ± 948.4 kcal; protein, 76.2 ± 26.1 g; fat, 89.6 ± 45.8 g; carbohydrate, 313.9 ± 112.5 g; omega3 fatty acid, 2.3 ± 1.0 g) before the intake of supplements. The nutrition status of these parameters had not changed during the experimental period.

### Eccentric contractions

**Fig. 1:**
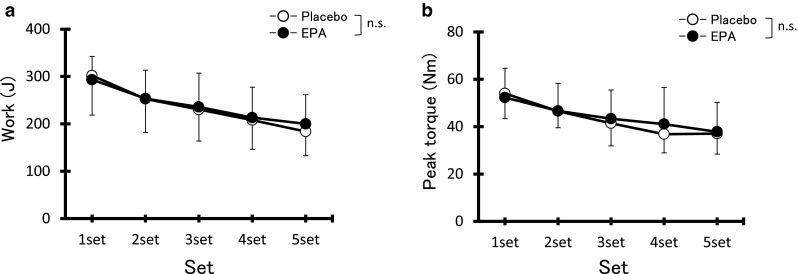
Changes (mean ± SD) in total work (**a**) and peak torque (**b**) over five sets of six maximal eccentric contractions in the placebo and EPA groups. *n.s.* not significant

No significant difference in work output (Fig. 1a) or peak torque (Fig. 1b) during any set of ECC was observed between the two groups.

### Maximal voluntary isometric contraction torque

**Fig. 2:**
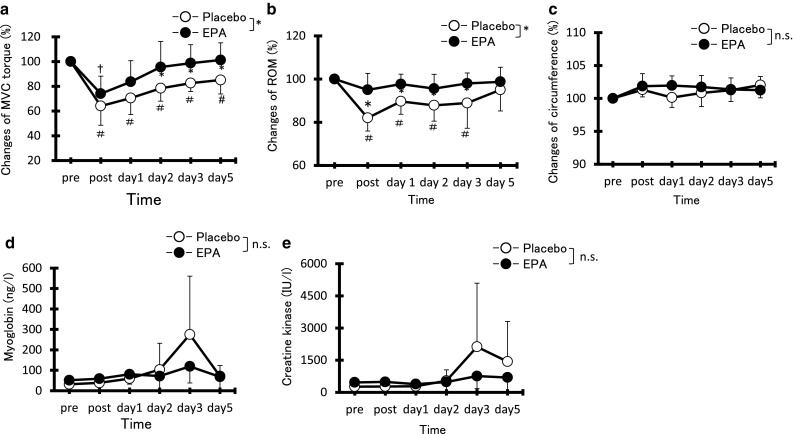
Changes (mean ± SD) in maximal voluntary isometric contraction (MVC) torque (**a**), range of motion (ROM) (**b**), circumference (**c**), myoglobin (**d**), and creatine kinase (**e**) before (pre), immediately after (post), and 1, 2, 3, and 5 days after eccentric contractions in the placebo group and EPA group. *p < 0.05 a significant difference between groups, ^†^ *p* < 0.05 a significant difference from pre-exercise value in the EPA group, ^#^ *p* < 0.05 a significant difference from pre-exercise value in the placebo group. *n.s.* not significant

MVC torque value was indicated by relative change compared with pre ECC value as 100 % (EPA group, 58.6 ± 14.9 Nm; PL group, 58.0 ± 11.6 Nm). MVC torque (Fig. 2a) in the PL group significantly decreased from immediately after to 5 days after exercise compared with pre-exercise levels (post, day 1, day 2, day 3, and day 5, *p* < 0.05). MCV torque in the EPA group decreased immediately after exercise compared with pre-exercise levels (*p* < 0.05). MVC was significantly higher in the EPA group than in the PL group at 2, 3, and 5 days after exercise [day 2; PL 78.4 ± 10.4 %, EPA 95.5 ± 20.1 %, Cohen’s *d* (*d*) = 1.04, 95 % confidence interval (CI) 0.15–1.85, day 3; PL 82.6 ± 6.8 %, EPA 98.7 ± 15.0 %, *d* = 1.39, CI 0.45–2.22, and day 5; PL 85.1 ± 11.4 %, EPA 101.3 ± 14.0 %, *d* = 1.27, CI 0.35–2.10, *p* < 0.05].

### Range of elbow motion

Figure 2b showed the values of ROM by relative change compared with pre ECC value as 100 % (EPA group, 127.3° ± 7.5°; PL group, 130.6° ± 10.4°). Although no significant decrease in elbow ROM from pre-exercise values was observed in the EPA group, a significant decrease in the PL group was observed immediately after exercise (reduction of 18.0 ± 6.1 %, *p* < 0.05) and remained lower than baseline levels at 1, 2, and 3 days (*p* < 0.05). Elbow ROM in the EPA group was significantly greater than that in the PL group from immediately after exercise to 3 days after exercise (post; PL 84.0 ± 7.2 %, EPA 96.4 ± 10.9 %, *d* = 1.88, CI 0.87–2.77, day 1; PL 89.0 ± 6.3 %, EPA 99.0 ± 8.1 %, *d* = 1.50, CI 0.55–2.35, day 2; PL 88.0 ± 7.1 %, EPA 97.6 ± 8.8 %, *d* = 1.11, CI 0.22–1.93, and day 3; PL 88.5 ± 10.9 %, EPA 99.6 ± 6.0 %, *d* = 1.01, CI 0.13–1.83, *p* < 0.05). ROM was also evaluated as absolute values in degrees. At post-ECC, days 2 and 3, elbow ROM degree was significantly different between the two groups. The degree of ROM indicated 111.5° ± 14.6° vs 120.3° ± 13.2° at post-ECC, 116.4° ± 10.1° vs 121.6° ± 9.5° at day 2, and 116.7° ± 11.7° vs 125.0° ± 7.2° at day 3 (PL group vs EPA group, respectively).

### Upper arm circumference

Upper arm circumference value was indicated by the change rate compared with the pre-ECC value as 100 % (EPA group, 25.0 ± 1.5 cm; PL group, 26.0 ± 1.7 cm). No significant difference in the upper arm circumference from pre-exercise values was observed in either group (EPA, 101.8 ± 1.1 %; PL, 101.3 ± 1.9 %). No significant difference between the two groups was observed at any time point (Fig. 2c).

### Muscle soreness

**Fig. 3:**
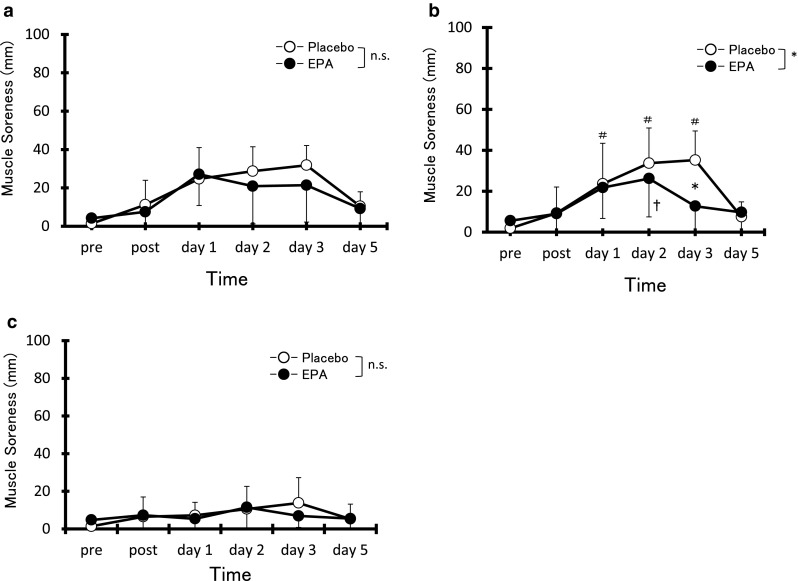
Changes (mean ± SD) in muscle soreness assessed by visual analog scale (VAS) for palpation of biceps brachii (**a**), brachialis (**b**) and brachioradialis (**c**) before (pre), immediately after (post), and 1, 2, 3, and 5 days after eccentric contractions in the placebo group and EPA group. *p < 0.05 a significant difference between groups, ^†^ *p* < 0.05 a significant difference from pre-exercise value in the EPA group, ^#^ *p* < 0.05 a significant difference from the pre-exercise value in the placebo group. *n.s.* not significant

Greater muscle soreness developed at the biceps brachii and brachialis at 1, 2, and 3 days after exercise compared with pre-exercise values in the PL group (Fig. 3a, b). In the EPA group, greater muscle soreness developed at the biceps brachii at 1, 2, and 3 days after exercise and at the brachialis at 2 days after exercise compared with pre-exercise values (Fig. 3a, b). Significantly greater muscle soreness at the brachialis was observed in the PL group compared with the EPA group at 3 days after exercise (PL, 34.2 ± 15.3 mm vs EPA, 12.7 ± 16.5 mm, *d* = 1.46, CI 0.52–2.31, *p* < 0.05). On the other hand, no significant increase in muscle soreness at the brachioradialis from pre-exercise values was observed in either group, with no significant difference between groups observed at any time point (Fig. 3c).

### Blood analyses

No significant differences in AA and DHA from pre-exercise values were observed in either group, with no significant differences between groups at any time point (data not shown). No significant difference in DGLA from pre-exercise values was observed in either group; however, significantly higher levels were observed in the PL group than the EPA group at 2 and 3 days after exercise (*p* < 0.05). On the other hand, EPA was significantly higher in the EPA group than in the PL group from before exercise to 3 days after exercise (pre; *d* = 0.52, CI 0.31–1.31, post; *d* = 0.50, CI 0.33–1.29, day 1; *d* = 0.80, CI 0.06–1.60, day 2; d = 0.65, CI 0.19–1.44, and day 3; *d* = 0.64, CI 0.20–1.44, *p* < 0.05).

**Fig. 4:**
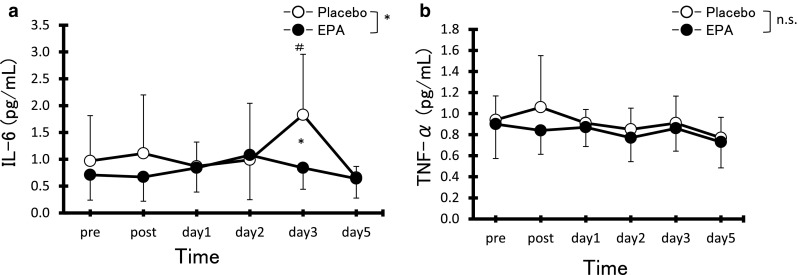
Changes (mean ± SD) in serum concentrations of IL-6 (**a**) and TNF-α (**b**) before (pre), immediately after (post), and 1, 2, 3, and 5 days after eccentric contractions in the placebo group and EPA group. *p < 0.05 a significant difference between groups, ^#^ *p* < 0.05 a significant difference from pre-exercise value in the placebo group. *n.s.* not significant

Although serum IL-6 levels in the PL group were significantly increased at 3 days after exercise compared with pre-exercise vales (Fig. 4a, *p* < 0.05), no significant difference in serum IL-6 levels after exercise compared with pre-exercise values was observed in the EPA group. Serum IL-6 levels were significantly higher in the PL group than the EPA group at 3 days after exercise (PL, 1.8 ± 1.1 μg/ml vs EPA, 0.8 ± 0.4 μg/ml, *d* = 1.17, CI 0.27–1.99, *p* < 0.05). In contrast, no significant difference in serum TNF-α levels after exercise compared with pre-exercise values was observed in either group, with no significant difference observed between groups (Fig. 4b).

Serum Mb levels in the PL group were significantly increased at 3 days after exercise compared with pre-exercise values (Fig. 2d, *p* < 0.05); however, no significant difference after exercise compared with pre-exercise values was observed in the EPA group. No significant differences in serum CK levels after exercise compared with pre-exercise values were observed in either group, with no significant difference observed between groups (Fig. 2e).

## Discussion

The present study evaluated the efficacy of 8 weeks of eicosapentaenoic and docosahexaenoic acid-rich fish oil supplementation in reducing muscle damage following ECC. The results demonstrate that the group with EPA and DHA acids-rich fish oil supplementation attenuated reductions of muscle torque loss and limited ROM. The supplementation also attenuated DOMS in brachialis and increased serum IL-6 levels at only day 3 after ECC. There were no changes in muscle swelling, DOMS in biceps brachii and brachioradialis, total work and peak torque during ECC, and muscle damage-related proteins. Especially, we first found the possibility that EPA and DHA supplementation could reduce the deficit in muscular strength after ECC. This result supports our original hypothesis.

No significant differences in peak torque or total work during ECC were observed between the EPA and PL groups. Twelve-week omega-3 polyunsaturated fatty acid supplementation (EPA: 2400 mg/day; DHA: 1200 mg/day) increased red blood cell (RBC) deformability as a consequence of incorporation of omega-3 polyunsaturated fatty acid into RBC membrane phospholipids (Andersson et al. 2002). Increased RBC deformability may lead to enhanced oxygen delivery to skeletal muscle with subsequent improvements in exercise performance (Mickleborough 2013). The result of the present study found no positive effects of omega-3 polyunsaturated fatty acid supplementation on the exercise performance. However, in a study on rats, an 8-week diet rich in DHA was associated with decreased fatigue during repetitive twitch contractions in vivo (Peoples and McLennan 2014). Data from animal studies suggest that omega-3 polyunsaturated fatty acids may play a beneficial role in improving the exercise performance. Further studies are required to investigate the effects of omega-3 polyunsaturated fatty acids on the magnitude of muscle fatigue produced by higher numbers of muscle contractions.

Our study first demonstrated that the possibility of EPA and DHA supplementation has a positive effect on MVC torque following eccentric exercise. The effects of EPA and DHA supplementation on strength loss after ECC have been controversial. Houghton and Onambele (2012) evaluated a resistance exercise for the lower limbs after 360 mg/day ingestion of EPA over 3 weeks and demonstrated no significant differences in muscle strength reduction between the EPA and placebo groups. In addition, DiLorenzo et al. (2014) showed that there was no effect on ECC-induced MVC reduction with 2000 mg/day DHA for 28 days and Lenn et al. (2002) reported no significant differences in muscle strength reduction with 400 mg/day EPA and 270 mg/day DHA for 30 days. The present study involved the ingestion of larger concurrent doses of EPA and DHA for twice the time as seen in Lenn’s study over a long period. Therefore, we suggested that ingestion of both 600 mg/day EPA and 260 mg/day DHA over an 8-week period would be necessary to show a positive effect for MVC loss after exercise.

Temporal strength losses following ECC are assumed to be due to excitation–contraction coupling failure (Warren et al. 1993). Although it was not possible to fully elucidate the mechanisms underlying this effect, we speculate that EPA and DHA supplementation has utility in protecting muscle cell membrane. Helge et al. (2001) reported that the total proportion of omega-3 polyunsaturated fatty acid in muscle cellular membrane was significantly increased after the ingestion of fish oil. Consumed omega-3 polyunsaturated fatty acids are incorporated into phospholipid, a major component of the cell membrane, and have been reported to inhibit the effects of inflammation and reactive oxygen species (Ling et al. 1998). Therefore, the incorporation of omega-3 polyunsaturated fatty acids into the muscle cell membrane alleviates tissue inflammation, thereby suppressing the disruption of neuromuscular junction and postsynaptic electrical transition.

The ROM limitation in the EPA group was significantly lower than that in the PL group in the present study. Since we speculate that EPA and DHA have a role in the protection of muscle cell membrane, it might attenuate the limited ROM, because the ROM following ECC has been attributed to inflammatory response within myofibrils leading to increases in passive stiffness (Chleboun et al. 1998). Tartibian et al. (2009) also demonstrated a decrease in ROM following EPA consumption compared with placebo. However, Lenn et al. (2002) reported that 30 days ingestion of EPA did not ameliorate limited ROM following ECC in elbow flexors. The present study conflicts with previous results reported by Lenn et al. (2002). Although the type of exercise and target muscle in the present study were the same as that of the previous study (Lenn et al. 2002), larger doses of EPA were administered over a longer period in the present study. Further studies are needed on the effect of EPA and DHA on ROM, but the amelioration of limited ROM in response to ECC may depend on the dose and duration of the supplement regimen in addition to the type of exercise.

The DOMS of the brachialis in the EPA group at 3 days after ECC was significantly lower than that in the PL group, but the DOMS of the biceps brachii did not change. Although the reason for the difference between two muscles is unclear, we suggest that the damage to ECC mainly occurred in the brachialis. The effect of EPA and DHA on DOMS remains controversial with previous studies arguing that there was a small number of subjects and large variability (DiLorenzo et al. 2014; Gray et al. 2014; Houghton and Onambele 2012; Jouris et al. 2011; Lenn et al. 2002; Tartibian et al. 2011). However, our result, which is limited in brachialis, demonstrates that EPA and DHA mitigate DOMS 3 days after ECC. The EPA and DHA supplementation alleviated damage in pain nerve endings, as discussed by a previous study (Cheung et al. 2003). In addition, since DOMS in brachioradialis did not change during experimental periods, brachioradialis might be a minor contributor in this ECC.

The present study demonstrated significantly lower increases in serum IL-6 levels at 3 days after exercise in the EPA group than in the PL group. Although no significant differences were observed between the two groups, there was a trend toward lower serum TNF-α levels and IL-6 immediately after exercise in the EPA group than in the PL group. These tendencies suggest that an inflammatory response has  occurred immediately after exercise and then anti-inflammatory response has occurred at 3 days. Otherwise, IL-6 has been demonstrated as a marker of inflammation with peak levels evident at 2 days after an eccentric exercise injury and a return to basal values 7 days after exercise (Phillips et al. 2003). In this stage, although we cannot conclude whether IL-6 is an inflammatory or anti-inflammatory response, we speculate that larger muscle damage had been induced in the PL group in comparison with the EPA group, because of the IL-6 activations of post-exercise and 3 days later.

Although serum levels of Mb, a marker of muscle damage, significantly increased in the PL group, no significant changes in Mb levels were observed in the EPA group, and there were no significant differences between the groups. Further, no significant differences in serum CK levels were observed between the two groups. Serum Mb and CK are known markers that indicate large differences in individuals (Clarkson et al. 1992). In the present study, the results of serum Mb and CK levels also showed large standard deviations, especially in the PL group. Although the present study was conducted in a parallel-group trial design because of “repeated bout effect” (Nosaka et al. 2005), the crossover design would have been more appropriate to minimize this effect. Therefore, these results were re-analyzed between the groups using logarithmic values of Mb and CK data because of heterogeneity of variance. This re-analysis indicated significant differences in serum Mb levels between the groups 3 days after ECC (data not shown). Although larger samples need to be used because of large individual differences, this result led us to suppose that EPA and DHA possess improvement on cellular membrane structure.

The time points of a significant difference between EPA and CON groups were different for each variable. We confirmed that no significant correlation between all muscle damage markers at each time point was observed in this study (data not shown). Hence, these markers such as MVC, DOMS, ROM, serum CK, and Mb are considered to be independent indicators of muscle damage. Regarding the time course of each parameter, we confirmed that decreases in MVC and ROM showed the peak at post-ECC, which was early phase, and relatively large effects with the EPA and DHA ingestion in comparison with the late phase phenomenon. These results are also similar to the previous studies (Chan et al. 2012; Chen et al. 2011). We assume that the EPA and DHA play a role in the protection of muscle cell membrane on the early phase. On the other hand, DOMS, CK, myoglobin, and IL-6 in the present study showed a peak at 3 days after ECC. These parameters were relatively small effects or there were no differences between the groups. We also speculate that EPA and DHA have little or indirect effect on the later phase.

Previous studies have reported the efficacy of either EPA or DHA or a combination of EPA and DHA supplementation on muscle damage (DiLorenzo et al. 2014; Houghton and Onambele 2012; Jouris et al. 2011; Lenn et al. 2002; Tartibian et al. 2011). Regarding DOMS, several papers including our study showed positive effects with EPA and DHA supplementation (Jouris et al. 2011; Tartibian et al. 2011). However, it has been shown that the ingestion of a single DHA (DiLorenzo et al. 2014) or EPA (Houghton and Onambele 2012) had no effect on DOMS. Although the dose and period are different from the previous studies (Jouris et al. 2011; Tartibian et al. 2011), we suggest that the ingestion of both EPA and DHA, especially at the ratio of approximately 2:1, has a synergistic effect on DOMS attenuation. On the other hand, IL-6 was attenuated by either single or simultaneous ingestion, but CK was decreased only by DHA and a combination of EPA and DHA (DiLorenzo et al. 2014; Houghton and Onambele 2012; Tartibian et al. 2009, 2011). Hence, we also speculate that EPA and DHA might have different roles, and thereby simultaneous ingestion of EPA and DHA has a possible synergistic effect.

There are three limitations in the present study. First, we examined only a single dose and duration regimen of EPA and DHA administration in a single-exercise model. Regarding the effect of EPA and DHA supplementation on muscle damage, the minimal effect was with 540 mg/day (EPA and DHA) according to Tartibian et al. (2009). Hence, we have determined the amount of supplementation in consideration of the minimal effect in addition to a physical burden of subjects. However, further investigation is needed to study the different doses of EPA and DHA to elucidate the appropriate amount. Second, both the amount and duration of the EPA and DHA regimen was different in previous studies. Therefore, it is unclear which the most important factor is (amount or period) in this study. Further study is required to investigate this point. Third, the degree of incorporated EPA and DHA in the blood is unclear because we did not measure the pre-supplementation of serum EPA and DHA. Hence, it is necessary to measure the serum EPA and DHA levels for understanding the blood inflow.

## Conclusion

In summary, the present study investigated the efficacy of 8 weeks EPA and DHA supplementation in reducing muscle damage following eccentric contractions. The results showed that 600 mg/day EPA and 260 mg/day DHA supplementation attenuated the reduction of muscle strength and limited ROM. Therefore, we concluded that supplementation with EPA and DHA has a positive effect for these two symptoms of muscle damage. Further studies may need to investigate the synergistic and/or single, time, and dose effects of the supplementation.

## Abbreviations

AA: Arachidonic acid
ANOVA: Analysis of variance
BCAA: Branched-chain amino acids
CK: Creatine kinase
DGLA: Dihomo-gamma-linolenic acid
DHA: Docosahexaenoic acid
DOMS: Delayed-onset muscle soreness
EPA: Eicosapentaenoic acid
IL-6: Interleukin-6
Mb: Myoglobin
MVC: Maximal voluntary isometric contraction
PL: Placebo
RBC: Red blood cell
ROM: Range of motion
RPE: Rating of perceived exertion
TNF-α: Tumor necrosis factor-alpha
VAS: Visual analog scale
